# The effect of oral sodium acetate administration on plasma acetate concentration and acid-base state in horses

**DOI:** 10.1186/1751-0147-49-38

**Published:** 2007-12-20

**Authors:** Amanda Waller, Michael I Lindinger

**Affiliations:** 1 Dept. of Human Health and Nutritional Sciences, University of Guelph, Guelph, ON, N1G 2W1, Canada

## Abstract

**Aim:**

Sodium acetate (NaAcetate) has received some attention as an alkalinizing agent and possible alternative energy source for the horse, however the effects of oral administration remain largely unknown. The present study used the physicochemical approach to characterize the changes in acid-base status occurring after oral NaAcetate/acetic acid (NAA) administration in horses.

**Methods:**

Jugular venous blood was sampled from 9 exercise-conditioned horses on 2 separate occasions, at rest and for 24 h following a competition exercise test (CET) designed to simulate the speed and endurance test of 3-day event. Immediately after the CETs horses were allowed water *ad libitum *and either: 1) 8 L of a hypertonic NaAcetate/acetic acid solution via nasogastric tube followed by a typical hay/grain meal (NAA trial); or 2) a hay/grain meal alone (Control trial).

**Results:**

Oral NAA resulted in a profound plasma alkalosis marked by decreased plasma [H^+^] and increased plasma [TCO_2_] and [HCO_3_^-^] compared to Control. The primary contributor to the plasma alkalosis was an increased [SID], as a result of increased plasma [Na^+^] and decreased plasma [Cl^-^]. An increased [A_tot_], due to increased [PP] and a sustained increase in plasma [acetate], contributed a minor acidifying effect.

**Conclusion:**

It is concluded that oral NaAcetate could be used as both an alkalinizing agent and an alternative energy source in the horse.

## Introduction

The administration of bicarbonate salts as alkalinizing agents has been extensively studied in both horses [1] and humans [2], and is of particular interest to the horseracing community where the testing of horses for illegal performance enhancing alkalinizing agents occurs. Sodium acetate (NaAcetate) has received some attention as an alkalinizing agent and possible alternative to sodium bicarbonate (NaHCO_3_) [3]. In contrast to NaHCO_3_, the ergogenic effects of NaAcetate do not appear to have been studied in horses, however a few studies have demonstrated its alkalinizing effect orally [3] and intravenously [4]. Of the limited studies of NaAcetate administration in horses, none have measured and reported the major plasma constituents describing acid-base status, therefore the magnitude and time course of variables that contribute to the origins of the metabolic alkalosis have been incompletely characterized; indeed the picture may be different from that obtained with NaHCO_3 _and other alkalinizing compounds. Identification of the origins of acid-base disturbances is facilitated by use of the physicochemical approach to acid-base status, which uses both dependent and independent variables to describe acid-base state. According to the physicochemical approach, the independent variables that determine plasma acid-base status are the concentration of strong ions in solution – defined as the strong ion difference ([SID]), the partial pressure of carbon dioxide (PCO_2_), and the concentration of weak acids in solution – defined as the total weak acid concentration ([A_tot_]). Thus the dependent acid-base variables – [H^+^], bicarbonate concentration ([HCO_3_^-^]) and total carbon dioxide concentration ([TCO_2_]) – only change when one or more of the independent variables are altered [5,6].

Acetate is produced naturally in the horse by hindgut fermentation, and of all the volatile fatty acids, acetate is produced in the greatest quantity [7]. Acetate is a metabolic precursor to acetyl-CoA, and tracer studies have shown that it is metabolized mainly to CO_2 _and water via the TCA cycle [8], generating ATP within the mitochondria. Indeed acetate appears to be an important energy source for the horse. Pethick et al. [9] found that acetate contributed up to 32% of total substrate oxidation in hindlimb muscles of horses at rest, while Pratt et al. [10] found that NaAcetate clearance was accelerated by exercise, suggesting that acetate may be used as an energy source during exercise as well. Thus supplemental acetate may be a practical alternative energy source for the horse, however the intestinal absorption and plasma electrolyte and acid-base effects of oral sodium acetate remain largely unknown. Therefore the purpose of the present study was to detail the time course and magnitude of the changes in key blood constituents that determine plasma acid-base state in horses, after oral administration of a sodium acetate/acetic acid solution, and to employ the physicochemical approach to describe the resulting acid-base disturbances. It was hypothesized that: 1) oral administration of a sodium acetate/acetic acid solution after prolonged submaximal exercise will result in a rapid and sustained increase in plasma [acetate]; and 2) a marked metabolic alkalosis will occur that is characterized by increased plasma [Na^+^] and elevated plasma [SID].

## Methods

### Animals

8 Standardbreds and 1 Thoroughbred (7 geldings, 2 mares; body weight 470 ± 11 kg; age 5–12 yrs) from the University of Guelph research herd were used. The study took place between November and February, and the horses underwent a 4–6 week diet and exercise acclimation/conditioning period during which they were housed in individual box stalls with 7 hrs of daily turnout in a half acre paddock with minimal forage available. Horses were exercise trained 4 days/week on a high speed treadmill (SATO, Sweden) and 2 days/week on an outdoor exerciser (Odyssey Performance Trainer, Campbellville, ON, Canada), until able to comfortably perform a competitive exercise test (CET) [11,12] on a high speed treadmill designed to significantly decrease muscle glycogen content [13] and result in total body water losses of 8–10 L [12]. The CET is designed to simulate the 2^nd ^day (speed and endurance test – classic format) of a one star CCI 3-day event, and includes the following phases: 10 min walk (1.7 m/s), 10 min trot (3.7 m/s), 2 min gallop (10.0 m/s), 20 min trot (3.7 m/s), 10 min walk (1.7 m/s), 8 min canter (8.0 m/s), and 30 min walk (1.7 m/s).

The horses were maintained on a diet consisting of sweet feed (Purina Check-R-Mix 12%, DCAB {Na^+ ^+ K^+ ^- Cl^-^} = -68.6 meq/kg) twice daily and mixed grass hay (DCAB = 303.2 meq kg^-1^) three times daily, with free access to water and a salt block. The amount of feed given was increased over this acclimation period such that during the final two weeks the horses were receiving 4 kg sweet feed and 6 kg hay daily (dietary DCAB = 154.5 meq kg^-1^), and there were no significant changes in the body masses of the horses during this time. The animal care and use procedures were approved by the University of Guelph Animal Care Committee and performed in accordance with the guidelines of the Canadian Council on Animal Care.

### Experimental Protocol

The study consisted of a treatment and control, thus each horse performed the CET twice in randomized order, separated by an 8–10 day interval [12,14] during which time exercise conditioning was maintained. On both sampling days beginning at 7 am, the hair coat over the jugular vein, 10–20 cm below the mandible, was clipped short to the skin on both sides of the neck. Each jugular vein catheterization site was aseptically prepared for insertion of catheters. A topical anesthetic, EMLA cream (2.5% lidocaine and 2.5% prilocaine; Astra Pharma, Mississauga, ON, Canada), was applied 25–30 min before insertion of catheters to desensitize the skin. Local anaesthetic (2% Xylocaine; Astra Pharma) was injected subcutaneously to complete the anesthesia. Catheters (14-gauge, 5.25 in; Angiocath, Becton-Dickinson, Mississagua, ON, Canada) were inserted anterograde into the left and right jugular veins, secured with tape and stitched to the skin. Four-way stopcocks with 50 cm extensions were attached to the catheters for ease of blood sampling. Patency of the catheters was maintained with sterile, heparinized 0.9% NaCl (2000 IU1^-1 ^NaCl).

A pre-exercise blood sample was taken at 8 am and then the CET was performed. Immediately upon completion of the final canter an 'end of exercise' blood sample was taken, following which the horse walked for 10 min. Upon cessation of exercise, the horse either 1) was nasogastically administered a NaAcetate-electrolyte solution consisting of 500 g NaAcetate (134 g Na & 366 g acetate), 250 ml acetic acid (250 g acetate), 32 g KCl, and 300 g glucose in 8 L of water (Osmolality = 2944 mOsm kg^-1^) (NAA trial), or 2) stood in stocks for equivalent amount of time (Control trial). The acetate + glucose dosage was calculated to be able to replace all the skeletal muscle glycogen glucosyl units degraded during the period of exercise, assuming a ~40% decrease in muscle glycogen content [13]. The amount of NaAcetate and acetic acid given was established by a series of 4 pilot studies to determine the maximum amounts tolerated without gastrointestinal upset, and then the amount of glucose needed to replace the remaining glycosyl units was calculated.

Within 20 minutes of cessation of exercise, the horses were given 2 kg sweet feed and 3 kg hay (0 min of recovery), with access to water *ad libitum*. Horses were given 2 kg sweet feed and 3 kg hay at 6 hrs of recovery, and 2 kg hay at 12 hrs recovery. Blood samples were taken at 20–60 min intervals up to 8 hrs of recovery, and again at 24 hrs of recovery, and horses remained in their stalls for the duration of sampling.

### Sample Analysis

Each blood sample was collected into7 ml heparinized vacutainers and immediately analyzed for plasma pH, the partial pressures of carbon dioxide (pCO_2_) and oxygen (pO_2_), and the plasma concentrations of Na^+^, Cl^-^, K^+^, Ca^2+^, lactate^- ^using a Nova Stat Profile 9^+ ^(NOVA Biomedical, Waltham, MA). Hematocrit (hct) was measured by conductivity and [HCO_3_^-^] and total carbon dioxide (TCO_2_) concentration were calculated using the Henderson-Hasselbach equation by the Nova Stat Profile 9^+^. It is important to note that the Nova may yield [HCO_3_^-^] and TCO_2 _values 2–3 mmoles/L higher than the instruments used (typically a Beckman model analyzer) by horse racing jurisdications [15]. Remaining blood was transferred into two 1.5 ml conical centrifuge tubes and centrifuged for 5 min at 15000 g to separate the plasma. Plasma protein concentration ([PP]) was determined (coefficient of variation (CV 0.83%) by using refractometry (Atogo clinical refractometer model SPR-T2; Atago, Tokyo, Japan). Plasma [acetate] was measured in duplicate spectrophotometrically using a commercially available kit (R-Biopharm, Marshall MI) (CV 1.6%).

### Calculations

Plasma [H^+^] was calculated using the measured pH such that:

pH = -log [H^+^]

Plasma Strong Ion Difference ([SID]) was calculated as the sum of the plasma concentrations of the strong cations minus the strong anions [5], such that:

[SID] = [Na^+^] + [K^+^] - [Cl^-^] - [lactate^-^]

In practice, the concentrations of the divalent cations and anions (Ca^2+^, Mg^2+^, PO_4_^2- ^and SO_4_^2-^) are small and the sum of their charges close to zero and can be ignored [6].

The plasma concentration of weak ions ([A_tot_]) was calculated as:

[A_tot_] = 2.04 * [PP] (g dL^-1^) + Ac (mmol L^-1^)

where 2.04 * [PP] was taken from Constable [16] based on formulas for estimating [A_tot_] in equine plasma with known albumin and globulin concentrations. Ac is the plasma acetate contribution to [A_tot_], calculated using AcidBasics II software (^©^2003, PD Watson) for each horse at each time point using measured values for [SID], pCO_2 _and [PP], and a K_A _for acetate of 1.74 × 10^-5 ^(eq L^-1^).

**Figure 1:**
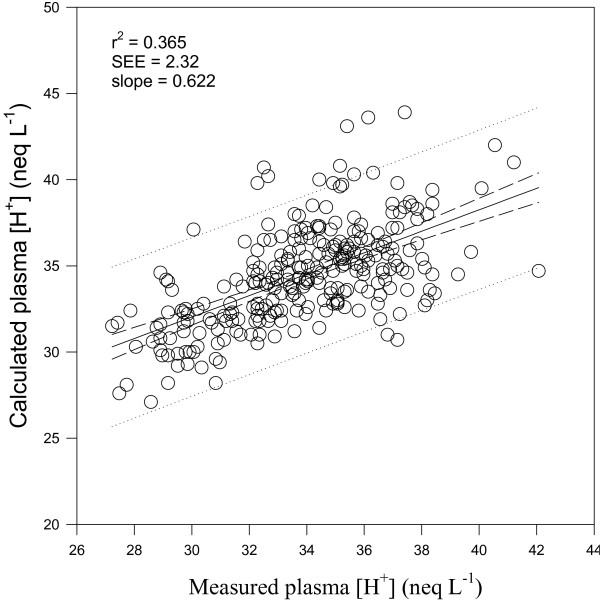
Linear regression relationship (solid lines) and 95% confidence interval (dashed lines) between measured and calculated plasma [H^+^] in 9 horses before and after a Competition Exercise Test. The exercise recovery period begins at 0 min, after horses were either given a hypertonic sodium acetate/acetic acid solution (NAA trial; solid square), or stood in stocks (Control trial; hollow circle). Provide the equation with the p value SEE = standard error of the estimate.

Calculations of dependent acid-base parameters (pH, [H^+^], [HCO_3_^-^], TCO_2_) were made using AcidBasics II software using the equation:

[H^+^] + (K_A _+ [SID]) [H^+^]^3 ^+ {K_A _([SID] - [A_tot_]) - (K_C _* pCO_2 _+ K_W_)} [H^+^]^2 ^- {K_A _(K_C _* pCO_2 _+ K_W_) + (K_3 _* K_C _* pCO_2_)} [H^+^] - K_A _* K_3 _* K_C _* pCO_2_) = 0

where K_W_, K_A_, K_3 _and K_C _are the equilibrium constants for dissociations of water, weak acids, carbonic acid and bicarbonate, respectively (K_W _= 4.4 * 10^-14 ^(eq L^-1^), K_A _= 3.9 * 10^-8 ^(eq L^-1^), K_3 _= 5.76 * 10^-11 ^(eq L^-1^), K_C _= 2.45 * 10^-11 ^(eq L^-1^)^2 ^mmHg^-1^. K_A _is the equilibrium constant for dissociation of weak acids in equine plasma in the present study, determined by linear regression analysis of measured vs calculated [H^+^]. (See Fig. 1; r^2 ^= 0.365, SEE = 2.32, slope = 0.622)

The contributions of the independent variables [A_tot_] and PCO_2 _to the dependent variable [H^+^] were determined by holding two of either [SID], pCO_2_, or [A_tot_] constant while calculating [H^+^] in response to changes in the third independent variable [17]. The contribution of the [SID] to the changes in [H^+^] was calculated by determining the contributions of pCO_2 _and [A_tot_], and then subtracting these from the measured change in [H^+^].

Total body water loss during the CET was determined as the change in body mass after accounting for fecal losses. Plasma osmolality was calculated according to the formula of Brownlow and Hutchins [18] for equine plasma modified to include plasma [acetate] such that:

Osmolality (mOsm kg^-1^) = 1.86([Na^+^] + [K^+^]) + [Glucose] + [Lactate^-^] + [acetate] + 9

### Statistics

Data are presented as mean ± standard error. Changes over time were assessed by one-way repeated measures analysis of variance. Differences between treatments were assessed by two-way repeated measures analysis of variance. When a significant F-ratio was obtained, means were compared using the all pairwise multiple comparison procedure of Holm-Sidak. Statistical significance was accepted when P < 0.05 at a power of 0.8.

## Results

Ambient temperature and humidity during the CET were 20.5 ± 0.2°C and 40.5 ± 3.0%, respectively. Total body water loss during the CET was 8.3 ± 0.3 L.

### Independent variables and electrolytes

**Figure 2:**
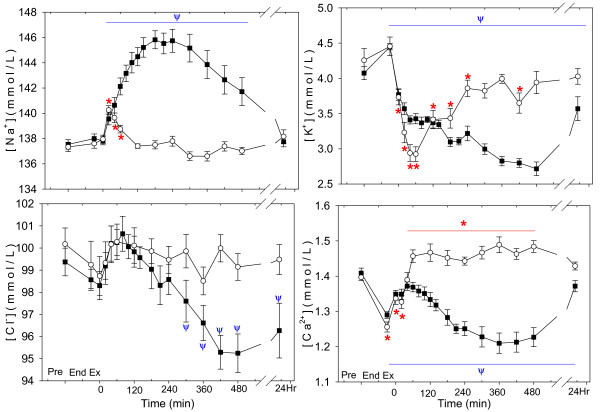
The time course of plasma electrolyte concentrations: (A) sodium (Na^+^), (B) potassium (K^+^), (C) chloride (Cl^-^) and (D) calcium (Ca^2+^) after a Competition Exercise Test. The exercise recovery period begins at 0 min, after horses were either given a hypertonic sodium acetate/acetic acid solution (NAA trial; solid square), or stood in stocks (Control trial; hollow circle). Horses were fed immediately after the 0 and 360 min samples. Values are mean ± SE for 9 horses. *, Ψ: significantly different from baseline (pre-exercise) time point for Control and NAA trials, respectively.

Plasma [Na^+^] (Fig. 2a) was increased from pre-exercise from 20–480 min and 20–60 min of recovery in the NAA and Control trials, respectively, with a significant difference between treatments (P <0.001). Plasma [K^+^] (Fig. 2b) was decreased from 0–240 min of recovery in the Control trial, and from 0 min to the end of sampling in the NAA trial, with no difference between treatments (P = 0.292). Plasma [Cl^-^] (Fig. 2c) was decreased from pre-exercise from 300 min to the end of sampling in the NAA trial, and did not differ from pre-exercise in the Control trial, with a significant difference between treatments (P <0.001). Plasma [Ca^2+^] (Fig. 2d) was decreased from pre-exercise from the end of exercise to 20 min of recovery, and increased from pre-exercise 60–480 min of recovery in the Control trial. Plasma [Ca^2+^] in the NAA trial was decreased from pre-exercise throughout the recovery period from the end of exercise to the end of sampling. There was a significant difference between trials (P <0.001).

Plasma [lactate] was increased from the end of exercise to 0 min of recovery and 100 min of recovery in the Control and NAA trials, respectively, with no differences between trials (P = 0.858) (Table 1).

**Table 1:** Jugular vein plasma pH, bicarbonate and lactate concentrations, at rest and during recovery from a Competition Exercise Test, after horses were either given 1) an oral sodium acetate/acetic acid solution followed by a typical feeding protocol (NAA trial), or 2) a typical feeding protocol alone (Control trial)

**Time (min)**	**pH**		**[HCO_3_^-^]**		**[Lactate]**	
	NAA	Control	NAA	Control	NAA	Control

Pre-Ex	7.48 ± 0.01	7.48 ± 0.01	36.5 ± 0.6	36.4 ± 0.9	0.7 ± 0.2	0.8 ± 0.2
End Ex	7.52 ± 0.01*	7.53 ± 0.01*	33.56 ± 0.4*	34.7 ± 0.8	2.8 ± 0.6*	2.6 ± 0.6*
0'	7.47 ± 0.01	7.48 ± 0.01	36.4 ± 0.8	35.9 ± 0.9	1.6 ± 0.1*	1.7 ± 0.3*
20'	7.47 ± 0.02	7.48 ± 0.01*	33.7 ± 1.4*	38.0 ± 0.9*	1.5 ± 0.2*	1.1 ± 0.3
40'	7.44 ± 0.01*	7.45 ± 0.01*	33.16 ± 1.2*	36.2 ± 1.1	1.5 ± 0.2*	1.2 ± 0.3
60'	7.43 ± 0.01*	7.44 ± 0.01*	33.8 ± 1.0*	34.4 ± 0.5	1.5 ± 0.2*	1.1 ± 0.3
80'	7.44 ± 0.01*		34.5 ± 1.2		1.4 ± 0.2*	
100'	7.44 ± 0.01*		34.8 ± 0.8		1.4 ± 0.2*	
120'	7.45 ± 0.01*	7.44 ± 0.1*	36.8 ± 0.8	34.3 ± 0.6*	1.2 ± 0.2	1.1 ± 0.2
140'	7.45 ± 0.01*		38.0 ± 0.8		1.2 ± 0.2	
180'	7.46 ± 0.01	7.46 ± 0.01*	37.8 ± 1.1	33.7 ± 0.4*	1.1 ± 0.2	1.3 ± 0.2*
210'	7.47 ± 0.01		39.7 ± 1.0*		1.1 ± 0.2	
240'	7.49 ± 0.01	7.46 ± 0.01*	40.46 ± 0.9*	34.9 ± 0.6	1.1 ± 0.2	1.1 ± 0.2
300'	7.51 ± 0.01*	7.46 ± 0.01*	42.01 ± 1.0*	34.2 ± 0.6*	1.2 ± 0.1*	1.0 ± 0.3
360'	7.51 ± 0.01*	7.45 ± 0.01*	44.1 ± 0.8*	33.8 ± 0.5*	1.1 ± 0.1	1.2 ± 0.3
420'	7.53 ± 0.01*	7.45 ± 0.01*	44.8 ± 0.9*	34.4 ± 0.4*	1.0 ± 0.2	0.8 ± 0.2
480'	7.53 ± 0.01*	7.46 ± 0.01*	44.5 ± 0.5*	34.0 ± 0.2*	0.9 ± 0.1	0.9 ± 0.2
24 h	7.49 ± 0.01	7.46 ± 0.01*	39.3 ± 1.5*	34.2 ± 0.7	1.2 ± 0.2	0.9 ± 0.2

Values are mean ± SE in mmol/l. * Significantly different (P < 0.05) from pre-exercise time point.

**Figure 3:**
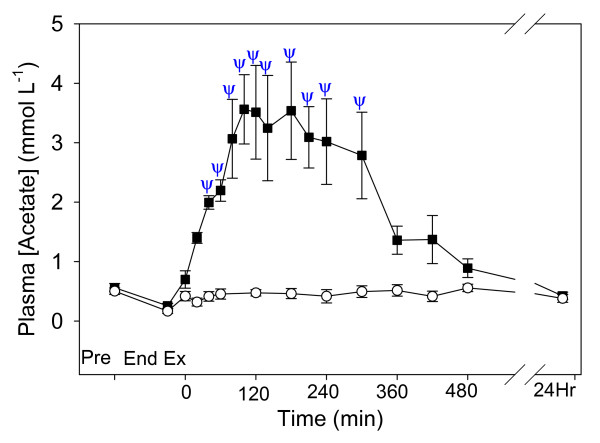
The time course of plasma acetate concentration after a Competition Exercise Test. The exercise recovery period begins at 0 min, after horses were either given a hypertonic sodium acetate/acetic acid solution (NAA trial; solid square), or stood in stocks (Control trial; hollow circle). Horses were fed immediately after the 0 and 360 min samples. Values are mean ± SE for 9 horses. *, Ψ: significantly different from baseline (pre-exercise) time point for Control and NAA trials, respectively.

Plasma [acetate] in the NAA trial was increased from pre-exercise (0.56 ± 0.07 mmol L^-1^) from 40–300 min of recovery and reached a maximum of 3.56 ± 0.58 mmol L^-1 ^at 100 min of recovery (Fig. 3). Plasma [acetate] was not different from pre-exercise in the Control trial, and there was a significant difference between trials (P = 0.002).

**Figure 4:**
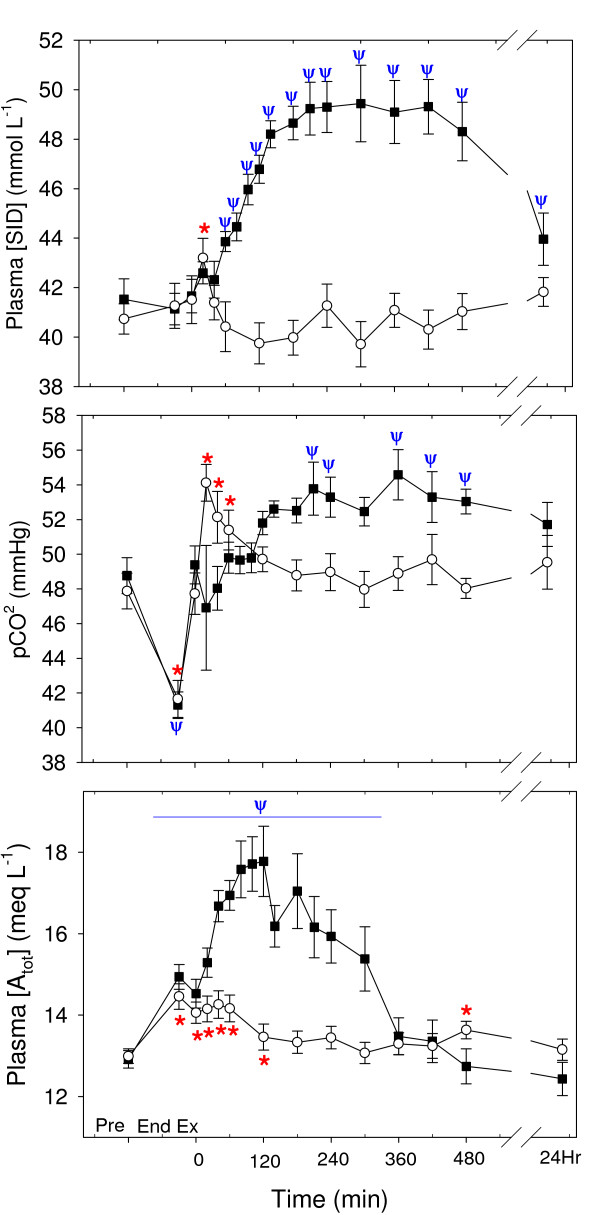
The time course of the independent acid-base variables in plasma: (A) strong ion difference ([SID]), (B) partial pressure of carbon dioxide (PCO_2_), and (C) total weak acid concentration ([A_tot_]), after a Competition Exercise Test. The exercise recovery period begins at 0 min, after horses were either given a hypertonic sodium acetate/acetic acid solution (NAA trial; solid square), or stood in stocks (Control trial; hollow circle). Horses were fed immediately after the 0 and 360 min samples. Values are mean ± SE for 9 horses. *, Ψ: significantly different from baseline (pre-exercise) time point for Control and NAA trials, respectively.

The time course of changes in independent acid-base variables are shown in Fig. 4. Plasma [SID] was increased from pre-exercise at 20 min of recovery in the Control trial, and from 60 min to the end of sampling in the NAA trial, with a significant difference between trials (P = 0.005). Plasma PCO_2 _(Fig. 4b) was decreased at the end of exercise in both trials, and increased from 20–60 min and 210–480 in the Control and NAA trials, respectively, with a trend towards an increased PCO_2 _in the NAA trial (P = 0.071). Plasma [A_tot_] (Fig. 4c) was increased from the end of exercise to 120 min of recovery and 300 min of recovery in the Control and NAA trials, respectively, with a significant difference between trials (P = 0.009).

### Dependent variables

**Figure 5:**
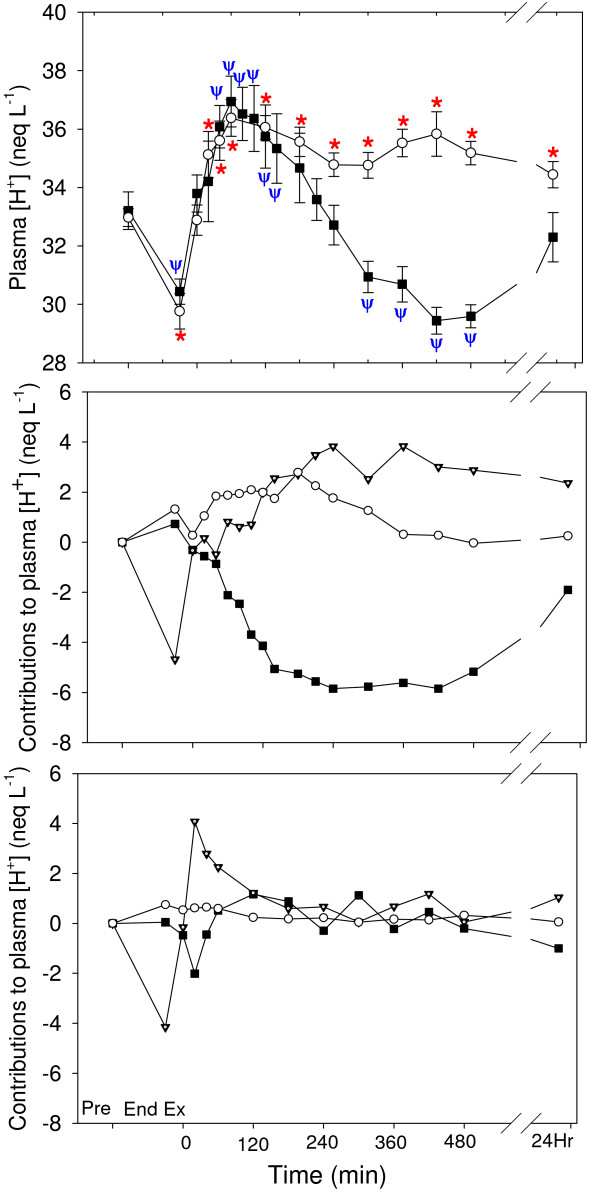
The time course of the dependent acid-base variable plasma hydrogen ion concentration ([H^+^]) after a Competition Exercise Test, and the contributions of the independent variables ([SID], solid square), total weak acid concentration ([A_tot_], hollow circle) or partial pressure of carbon dioxide (PCO_2_, solid triangle) to the change in plasma [H^+^], for (B) the NAA trial and (C) the Control trial. The exercise recovery period begins at 0 min, after horses were either given a hypertonic sodium acetate/acetic acid solution (NAA trial; solid square), or stood in stocks (Control trial; hollow circle). Horses were fed immediately after the 0 and 360 min samples. Values are mean ± SE for 9 horses. *, Ψ: significantly different from baseline (pre-exercise) time point for Control and NAA trials, respectively.

Plasma [H^+^] (Fig. 5a) in the NAA trial was increased from 40–180 min of recovery, and decreased from 300–480 min of recovery. Plasma [H^+^] in the Control trial was increased from pre-exercise from 20 min of recovery to the end of sampling. There was a significant difference between trials (P = 0.006). The contributions of the independent variables to the change in plasma [H^+^] are shown in Fig. 5b and Fig. 5c. The main contributor to the decreased [H^+^] at the end of exercise in both trials was the decreased PCO_2_. The decreased [H^+^] during the latter recovery period of the NAA trial (Fig. 5b) was entirely due to increased [SID], while increases in PCO_2 _and [A_tot_] contributed an acidifying effect.

**Figure 6:**
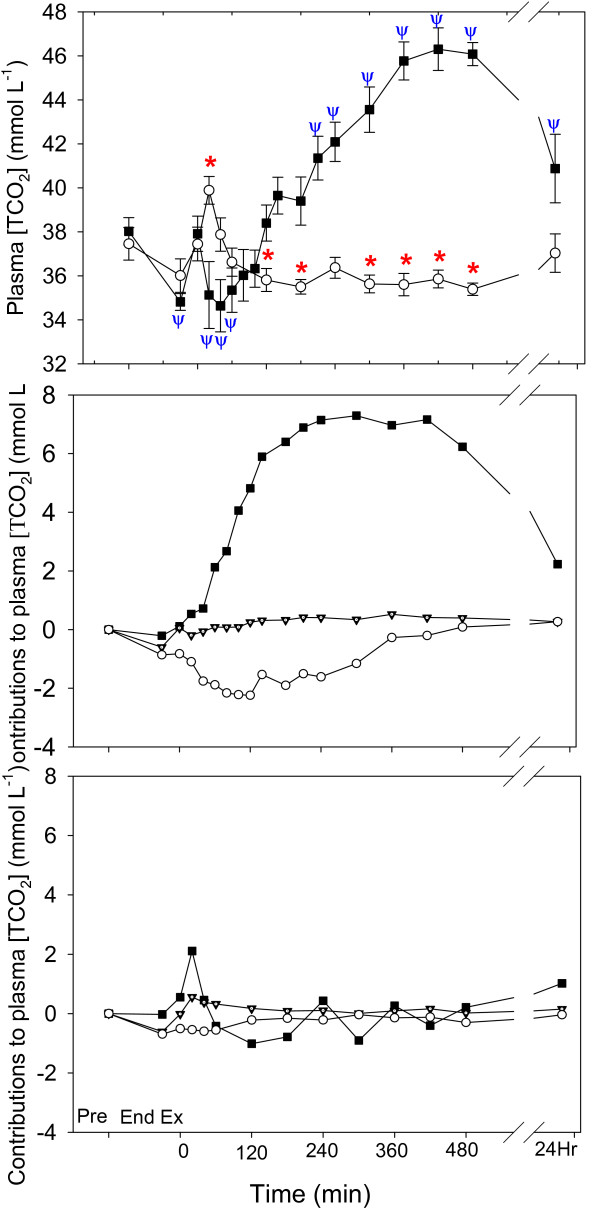
The time course of the plasma total carbon dioxide concentration ([TCO_2_]) after a Competition Exercise Test, and the contributions of the independent variables ([SID], solid square), total weak acid concentration ([A_tot_], hollow circle) or partial pressure of carbon dioxide (PCO_2_, solid triangle) to the change in [TCO_2_], for (B) the NAA trial and (C) the Control trial. The exercise recovery period begins at 0 min, after horses were either given a hypertonic sodium acetate/acetic acid solution (NAA trial; solid square), or stood in stocks (Control trial; hollow circle). Horses were fed immediately after the 0 and 360 min samples. Values are mean ± SE for 9 horses. *, Ψ: significantly different from baseline (pre-exercise) time point for Control and NAA trials, respectively.

Plasma [TCO_2_] (Fig. 6a) in the NAA trial was decreased at the end of exercise and 20–60 min of recovery, and increased from 210–480 of recovery. Plasma [TCO_2_] in the Control trial was increased at 20 min of recovery and decreased at 120, 180 and 300–480 min of recovery. There was a significant difference between trials (P < 0.001). The contributions of the independent variables to the change in plasma [TCO_2_] are shown in Fig. 6b and Fig. 6c. The increased [TCO_2_] in the latter recovery period of the NAA trial (Fig. 6b) was entirely due to increases in [SID], while an increased [A_tot_] contributed an acidifying effect.

Plasma pH and [HCO_3_^-^] (Table 1) are shown to provide terms of reference with respect to the majority of the acid-base literature. Plasma pH in the NAA trial was decreased from 40–180 min of recovery, and increased from 300–480 min of recovery. Plasma pH in the Control trial was decreased from pre-exercise from 20 min of recovery to the end of sampling, and there was a significant difference between trials (P = 0.004). Plasma [HCO_3_^-^] in the NAA trial was decreased at the end of exercise and 20–60 min of recovery, and increased from 210–480 of recovery. Plasma [HCO_3_^-^] in the Control trial was increased at 20 min of recovery and decreased at 120, 180 and 300–480 min of recovery. There was a significant difference between trials (P < 0.001).

### Plasma osmolality, protein and water consumption

**Figure 7:**
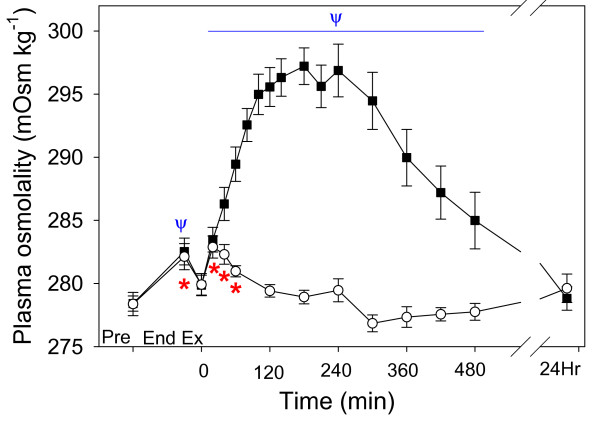
The time course of the changes in plasma osmolality, after a Competition Exercise Test. The exercise recovery period begins at 0 min, after horses were either given a hypertonic sodium acetate/acetic acid solution (NAA trial; solid square), or stood in stocks (Control trial; hollow circle). Horses were fed immediately after the 0 and 360 min samples. Values are mean ± SE for 9 horses. *, Ψ: significantly different from baseline (pre-exercise) time point for Control and NAA trials, respectively.

**Figure 8:**
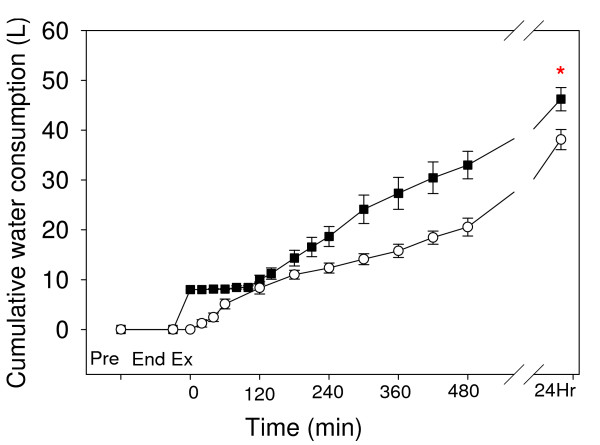
The time course of cumulative water consumption, after a Competition Exercise Test. The exercise recovery period begins at 0 min, after horses were either given a hypertonic sodium acetate/acetic acid solution (NAA trial; solid square), or stood in stocks (Control trial; hollow circle). Horses were fed immediately after the 0 and 360 min samples. Values are mean ± SE for 9 horses. * = significantly different from the Control trial.

Calculated plasma osmolality (Fig. 7) was increased at the end of exercise in both trials, and from 20–480 min and 20–60 min of recovery in the Acetate and Control trials, respectively. There was a significant difference between trials (P < 0.001). Horses consumed significantly more water in the NAA trial such that total water consumption (including the 8 L given nasogastrically) at 24 h of recovery (Fig. 8) was 46.2 ± 2.3 L and 38.1 ± 2.0 L in the Acetate and Control trials, respectively (P = 0.043).

[PP] was increased from the end of exercise to 120 min and 240 min of recovery in the Control and NAA trials, respectively, with no difference between trials (P = 0.428) (Table 2). Hematocrit (hct) was increased at the end of exercise in both trials and from 0–20 min and 40–140 min of recovery in the Control and NAA trials, respectively, with no difference between trials (P = 0.175) (Table 2). Plasma PO_2 _was increased at the end of exercise in both trials, with no difference between trials (P = 0.301) (Table 2). Plasma [glucose] was increased from the end of exercise until 360 min of recovery in the Control trial and until the end of sampling in the NAA trial, with no difference between trials (P = 0.386) (Table 2).

**Table 2:** Jugular vein hematocrit, plasma glucose and protein concentrations, and PO_2 _at rest and during recovery from a Competition Exercise Test, after horses were either given 1) an oral sodium acetate/acetic acid solution followed by a typical feeding protocol (NAA trial), or 2) a typical feeding protocol alone (Control trial)

**Time (min)**	**Hct**		**[Glucose]**		**[PP]**		**PO_2_**	
	NAA	Control	NAA	Control	NAA	Control	NAA	Control

Pre-Ex	37.1 ± 0.6	37.3 ± 0.7	4.7 ± 0.1	4.7 ± 0.1	6.1 ± 0.1	6.2 ± 0.1	59.4 ± 7.9	69.6 ± 13.9
End Ex	49.4 ± 0.5*	50.2 ± 0.7*	5.6 ± 0.2*	5.2 ± 0.3*	7.2 ± 0.1*	7.2 ± 0.2*	128.8 ± 7.6*	150.2 ± 11.5*
0'	39.7 ± 0.6	39.2 ± 0.6*	5.2 ± 0.2*	5.2 ± 0.1*	6.8 ± 0.1*	6.7 ± 0.2*	75.4 ± 11.6	77.9 ± 9.2
20'	40.3 ± 0.8*	38.7 ± 0.6*	5.5 ± 0.2*	5.5 ± 0.1*	7.0 ± 0.2*	6.9 ± 0.2*	93.9 ± 21.*	38.3 ± 2.1*
40'	41.1 ± 1.1*	38.2 ± 0.7	5.9 ± 0.1*	6.6 ± 0.1*	7.2 ± 0.2*	6.9 ± 0.2*	71.5 ± 14.3	45.2 ± 6.7*
60'	41.9 ± 0.7*	38.2 ± 0.8	6.1 ± 0.2*	7.1 ± 0.2*	7.2 ± 0.2*	6.8 ± 0.2*	41.8 ± 3.4	47.6 ± 11.0*
80'	41.3 ± 0.6*		6.5 ± 0.3*		7.1 ± 0.1*		43.5 ± 3.2	
100'	41.1 ± 0.7*		6.8 ± 0.3*		7.0 ± 0.2*		51.4 ± 11.9	
120'	40.9 ± 0.8*	37.5 ± 1.3	6.8 ± 0.4*	6.9 ± 0.4*	7.0 ± 0.2*	6.5 ± 0.2*	38.6 ± 1.6	75.2 ± 19.9
140'	40.7 ± 0.8*		6.6 ± 0.4*		6.9 ± 0.2*		44.2 ± 3.6	
180'	39.3 ± 0.5	37.8 ± 0.9	6.2 ± 0.3*	6.0 ± 0.3	6.7 ± 0.1*	6.4 ± 0.2	50.6 ± 10.5	56.1 ± 2.5
210'	39.0 ± 0.3		5.9 ± 0.2*		6.5 ± 0.1*		44.1 ± 5.6	
240'	40.1 ± 0.8*	41.0 ± 1.2*	6.2 ± 0.3*	5.3 ± 0.1*	6.4 ± 0.1*	6.4 ± 0.1*	60.5 ± 8.6	48.8 ± 4.0*
300'	37.2 ± 0.8	36.2 ± 0.7	6.0 ± 0.3*	5.3 ± 0.1*	6.2 ± 0.2	6.2 ± 0.2	68.2 ± 14.8	50.4 ± 7.3
360'	36.8 ± 0.9	36.8 ± 0.4	5.8 ± 0.2*	5.3 ± 0.2*	5.9 ± 0.2	6.2 ± 0.2	75.0 ± 12.3	65.5 ± 10.2
420'	36.0 ± 1.0	37.5 ± 0.4	5.5 ± 0.2*	5.0 ± 0.2	5.9 ± 0.2	6.3 ± 0.2	39.6 ± 3.2	46.7 ± 7.1
480'	36.8 ± 1.0	38.2 ± 0.2	5.6 ± 0.2*	5.0 ± 0.1	5.8 ± 0.2	6.5 ± 0.1*	46.3 ± 3.8	49.6 ± 5.6
24 h	40.7 ± 1.1*	41.0 ± 0.4*	5.5 ± 0.2*	4.9 ± 0.1	5.9 ± 0.2	6.4 ± 0.1	80.2 ± 18.9	124.2 ± 34.1*

Hct = hematocrit, [PP] = [plasma protein]. Values are mean ± SE, with [Glucose] in mmol/l, Hct in %, [PP] in g/dl and pO_2 _in mmHg. * Significantly different (P < 0.05) from pre-exercise time point.

## Discussion

This study appears to be the first to detail the time course of acute changes in plasma dependent and independent acid-base variables after NaAcetate supplementation in horses. The nasogastric administration of a NaAcetate/acetic acid solution in 8 L of water, followed by a typical hay and grain meal, resulted in a profound plasma alkalosis marked by decreased plasma [H^+^] and increased plasma [TCO_2_] and [HCO_3_^-^] as compared to Control. The primary contributor to the plasma alkalosis was an increased [SID], as a result of increased plasma [Na^+^] and decreased plasma [Cl^-^]. An increased [A_tot_], due to increases in [PP] and a sustained increase in plasma [acetate], contributed a minor acidifying effect.

### Electrolytes and Independent Variables

Plasma [SID] in the NAA trial was increased from 60 min post exercise until the end of sampling, with a peak increase of 8 mmol L^-1 ^above the pre-exercise time point. The increase in plasma [SID] was primarily due to a large and sustained increase in plasma [Na^+^], secondary to intestinal absorption of the large sodium load given (~11.4% of total extracellular sodium) [1]. Based on the amount of Na^+ ^given in the solution, if complete and simultaneous distribution of the Na^+ ^in the entire ECFV (~100 L) occurred, the peak increase in plasma [Na^+^] could have been up to 58 mmol L^-1^. Thus the time course of plasma [Na^+^] in the present study represents the interactions between intestinal rate of Na^+ ^absorption, tissue uptake and renal Na^+ ^excretion. To date there appear to be no previous studies reporting the effects of oral NaAcetate on plasma electrolytes in horses, however similar increases in plasma [Na^+^] have been seen with comparable doses of NaHCO_3 _(500 to 750 g given nasogastrically in 3 L of water) [19]. In contrast, when a smaller amount of NaAcetate (110 g) was given intravenously (i.v.) the increase in plasma [Na^+^] was more rapid and less prolonged, occurring from 20–60 min post infusion [4].

Also contributing to the increased [SID] in the NAA trial of the present study was a decreased plasma [Cl^-^], which has been demonstrated previously with oral NaHCO_3 _administration in horses [20,21] and humans [2]. This decreased plasma [Cl^-^] is not likely a result of increased renal excretion of Cl^-^, as NaHCO_3 _ingestion in humans slightly increased renal tubular Cl^- ^absorption [22], as would be expected with increased HCO_3_^- ^delivery to the tubules. A dilutional effect of increased plasma volume as a result of increased plasma osmolality also does not appear to be the cause because [PP] and hct did not decrease with NaAcetate administration. However, increased extracellular osmolality results in rapid activation of volume regulatory mechanisms in both erythrocytes [23] and skeletal muscle [24,25]. Within these cells, shrinkage elicits a regulatory volume increase driven by an inwardly directed Na^+^-K^+^-2Cl^- ^cotransporter (NKCC) with coinciding increase in Na^+^-K^+^-ATPase activity [24,25]. The decreased plasma [Cl^-^] and [K^+^] in the NAA trial of the present study is consistent with increased activities of the NKCC and Na^+^-K^+^-ATPase. Short-term regulators of Na^+^-K^+^-ATPase activity include intracellular [Na^+^], extracellular [K^+^], plasma [insulin] and plasma catecholamines [26]. It has also been suggested that a NaHCO_3_-induced alkalosis increases intracellular [Na^+^] through exchange of intracellular H^+ ^via the Na^+^-H^+ ^antiporter, which would in turn activate the Na^+^-K^+^-ATPase [27] and result in decreased extracellular [K^+^]. Indeed NaHCO_3 _therapy is used clinically in treating horses with hyperkalemic periodic paralysis [28]. There was likely also a renal contribution to the decreased plasma [K^+^] in the present study, as alkalosis has been shown to stimulate Na^+^-K^+^-ATPase-mediated uptake of K^+ ^into the principal cells of the kidney, resulting in enhanced K^+ ^secretion [29], and NaHCO_3 _loading has been shown to result in increased renal excretion of K^+ ^in humans [30] and horses [20].

A rapid and prolonged decrease in plasma [Ca^2+^] also occurred in the present study, which is consistent with the results of previous equine studies using NaAcetate [4] or NaHCO_3 _[20,31], and likely caused by an increased binding of plasma proteins to ionized calcium secondary to the reduced plasma [H^+^] associated with the induced alkalosis [32]. Kline et al. [4] found that iv infusion of NaAcetate decreased serum [Ca^2+^] and [K^+^] to a greater extent than NaHCO_3_, suggesting that the neuromuscular problems that may occur in horses given large doses of NaHCO_3_[20,33], may also arise with NaAcetate administration. Indeed several horses in the present study briefly exhibited mild muscle fasciculations.

Several studies have demonstrated that venous PCO_2 _increases after oral NaHCO_3 _administration in resting horses [19-21], with larger doses ≥ 500 g inducing hypercapnia for at least 12 hours [19]. I.v. infusion of NaAcetate led to an increased PCO_2 _in humans [34], but not in horses [4]. In the present study, however, PCO_2 _was increased from 210–480 min of recovery in the NAA trial, likely due to the marked metabolic alkalosis initiating a hypoventilary response [35].

NaAcetate administration resulted in a larger and more prolonged increase in plasma [A_tot_], compared to Control, due to greater increases in [PP] and plasma [acetate]. [PP] was increased during the initial recovery period for both trials, which is expected to occur as a result of exercise-induced fluid shifts [36] and bi-directional flow of water and electrolytes between the blood and gastrointestinal tract during feeding [37]. However, the very hypertonic NaAcetate solution (2944 mOsm kg^-1^) would have resulted in a greater flux of low [ion] fluid into the gastrointestinal tract, resulting in the 8% greater increase in [PP] in the early recovery period of the NAA trial as compared to Control. As mentioned above, although plasma volume expansion could be expected with NaAcetate administration due to the large and prolonged increase in plasma osmolality [3,38], this does not appear to have occurred as there was no decrease in [PP]. It is likely that increased plasma osmolality resulted in intracellular fluid loss, but no net gain of extracellular fluid volume occurred due to a large intraluminal fluid shift. Additionally, horses in the NAA trial consumed significantly more water over the 24 h sampling period, however they generally did not begin consuming water until at least 120 min of recovery, which would have contributed to the plasma dehydration and increased [PP] up to 240 min of recovery.

To our knowledge this study appears to be the first study to measure plasma [acetate] after oral acetate administration. Plasma [acetate] was increased from 40–300 min post NaAcetate administration and reached a maximum of 3.56 ± 0.58 mmol L^-1 ^at 100 min of recovery in the NAA trial, contributing to the increased [A_tot_]. When 112 g NaAcetate was given intravenously [10], plasma [acetate] increased to ~16 mmol L^-1 ^by 2 min post infusion and remained increased until 40 min post infusion. The more sustained and smaller increase in plasma [acetate] with oral administration likely represents a steady state between intestinal absorption and tissue (primarily skeletal muscle) extraction. Acetate transport across lipid bilayer membranes may occur by free diffusion of the undissociated acid [39] and its transport is facilitated by plasma membrane monocarboxylate transporters (**MCT**s) [40], thus cellular uptake occurs rapidly. Indeed, based on the total amount of acetate given in the present study, if complete and simultaneous distribution in the ECFV (~100 L) occurred, the peak increase in plasma [acetate] could have been up to 102 mmol L^-1^. Thus the time course of plasma [acetate] in the present study implies that tissue extraction of absorbed acetate occurs rapidly, suggesting that oral acetate could be a potential alternate energy source for horses. This supports the findings of Pratt et al. [10] who found that when NaAcetate was given intravenously prior to exercise the acetate was cleared from the plasma more rapidly than at rest, suggesting that the acetate may have been used as an energy source for the working muscle. Clearly the supplementation of even small amounts of NaAcetate to racehorses prior to racing would be ill-advised due to the risk of exceeding the testing threshold for an alkalinizing agent, however acetate could still potentially be an effective post race or post training supplemental energy source. In the present study the effect of prior exercise on NaAcetate uptake and metabolism is unknown, and a potential future direction of study would be to compare the effects of post-exercise acetate administration vs resting acetate administration.

### Dependent Variables

Nasogastric administration of a hypertonic NaAcetate/acetic acid solution in 8 L of water, followed by a typical hay and grain meal, resulted in a profound plasma alkalosis marked by decreased plasma [H^+^] and increased plasma [HCO_3_^-^] and [TCO_2_]. When the physicochemical determinants of the dependent variables were quantified (Figs. 5b/c &6b/c), the primary contributor to the plasma alkalosis was an increased [SID] from 60 min post administration until the end of sampling, as a result of increases in plasma [Na^+^] and decreases in plasma [Cl^-^]. In contrast, an increased [A_tot_], due to increases in [PP] and a sustained increase in plasma [acetate], contributed a minor acidifying effect. Finally, despite a tendency towards increased PCO_2 _with NaAcetate supplementation, PCO_2 _had little effect on plasma [H^+^] or [TCO_2_].

This appears to be the first study to detail the effects of NaAcetate administration on plasma [TCO_2_] in horses and knowledge of the effects of NaAcetate supplementation on acid-base status is of practical interest to the racing community. A plasma [TCO_2_] testing threshold of greater than 37 mmol/l is used by many racing jurisdictions to determine whether a horse has been administered an alkalinizing agent for the purpose of performance enhancement (see the 2008 paper by Lindinger and Waller [6]). Lloyd and Rose [3] compared the effects of nasogastric administration of ~250 g NaAcetate or NaHCO_3 _in 2 L of water and found that plasma [HCO_3_^-^] (which is ~95% of [TCO_2_]) peaked at 8 h post NaAcetate administration with an average increase of 11.3 mmol/l from baseline, while the increase in [HCO_3_^-^] and time to peak for NaHCO_3 _administration was 7.6 mmol L^-1 ^and 5.5 h, respectively. The authors concluded that NaAcetate produced a greater metabolic alkalosis with a later peak alkalinizing effect than NaHCO_3_. In the present study, plasma [TCO_2_] was not increased until 210 min post NaAcetate administration, reaching a peak of 46.3 mmol L^-1 ^at 420 min (7 h) post administration (an increase of 8.3 mmol L^-1 ^above pre-exercise). Thus the results of the present and previous studies suggest that NaAcetate could be used as an alkalinizing compound for performance enhancement, and in fact the delay in peak metabolic alkalosis that appears to occur with NaAcetate administration – this may make detection of the alkalinizing agent more difficult when pre-race TCO_2 _testing is used. Interestingly, pre-exercise plasma [TCO_2_] for the NAA and Control trials in the present study was 38.0 ± 0.6 and 37.5 ± 0.7 mmol L^-1^, respectively, with these horses fed a typical racehorse diet. Accordingly, the results of this study show that some horses naturally demonstrate [TCO_2_] that may approach or exceed the testing threshold, even when no alkalinizing substances have been given.

### Usefulness and limitations of the physicochemical approach

The main advantage of the physicochemical approach is the ability to identify and quantify the origins of an acid-base disturbance. The knowledge of why changes in [H^+^] and [TCO_2_] occur enhances the understanding of acid-base physiology, and may be useful in developing alternative testing strategies to determine whether illegal alkalinizing agents have been given.

The calculation of [A_tot_] proposed for use in horses previously [16] assumes normal and constant plasma acetate and phosphate concentrations. Because acetate is a weak acid and plasma [acetate] was altered in the present study, it was necessary to include an acetate component in the calculation of [A_tot_]. The plasma acetate contribution to [A_tot_] in the present study was calculated using AcidBasics II software (^©^2003, PD Watson) for each horse at each time point using measured values for [SID], pCO_2 _and [PP], and a K_A _for acetate of 1.74 × 10^-5 ^(eq L^-1^). The contributions of the independent variables to changes in the dependent variables (pH, [H^+^], [HCO_3_^-^], TCO_2_) were also determined using AcidBasics II software, using an equilibrium constant for dissociation of weak acids (K_A_) determined by linear regression analysis of measured vs calculated [H^+^]. Some of the difference between measured and calculated [H^+^] in the present study is likely due to inherent inaccuracy in the new dissociation constant for [A_tot_] and the conversion factor used to produce [A_tot_] from plasma [protein] and plasma [acetate]. It appears that additional research using many more horses may be required to obtain accurate values for [A_tot_] when large changes in plasma [acetate] occur.

### Summary and conclusion

The present study quantified the magnitude and time course of the main physicochemical determinants of acid-base status in horses after post exercise oral NaAcetate. The nasogastric administration of a NaAcetate/acetic acid solution in 8 L of water, followed by a typical hay and grain meal, resulted in a profound plasma alkalosis marked by decreased plasma [H^+^] and increased plasma [TCO_2_] and [HCO_3_^-^] as compared to Control. The primary contributor to the plasma alkalosis was an increased [SID], as a result of increased plasma [Na^+^] and decreased plasma [Cl^-^], while an increased [A_tot_], due to increases in [PP] and a sustained increase in plasma [acetate], contributed a minor acidifying effect. It is concluded that oral NaAcetate could potentially be used as both an alkalinizing agent and an alternative energy source in the horse.

## Competing interests

The author(s) declare that they have no competing interests.
